# Intracranial Capillary Hemangioma in the Posterior Fossa of an Adult Male

**DOI:** 10.1155/2016/6434623

**Published:** 2016-09-22

**Authors:** Jordan Nepute, Jinping Lai, Yihua Zhou

**Affiliations:** Department of Radiology, Saint Louis University School of Medicine, 3635 Vista Boulevard, Saint Louis, MO 63110, USA

## Abstract

Intracranial capillary hemangioma (ICH) is a rare entity, with approximately 24 reported cases in the literature. There are only three reported cases of ICH in an adult male. In this case report, we describe the fourth documented case of ICH in an adult male and, to the best of our knowledge, the first ever documented case of ICH in the posterior fossa of an adult male. We also discuss its imaging appearance and differential diagnosis.

## 1. Case Presentation

A 40-year-old male with a history of hypertension and diabetes presented with a four-day history of right earache, headache, and vertigo. He was initially seen by his primary care physician and given antibiotics for an ear infection; however, when his vertigo worsened, he subsequently went to an outside hospital emergency department. After being diagnosed with hemorrhage in the posterior fossa, he was transferred to our institution.

**Figure 1:**
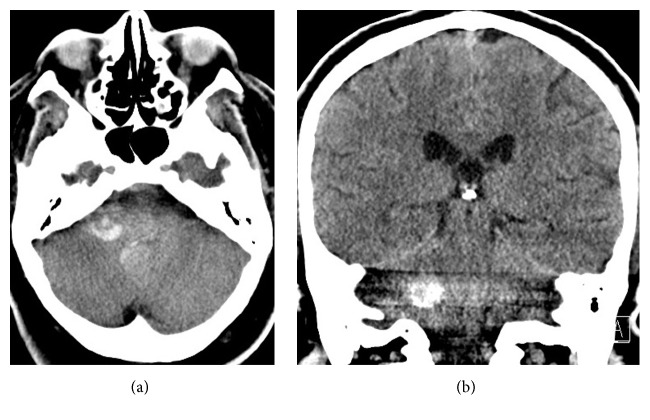
Axial (a) and coronal (b) noncontrast computed tomography (CT) of the head demonstrates hemorrhage in the region of the inferior right cerebellar peduncle, which appeared intraparenchymal, with extension into the fourth ventricle.

**Figure 2:**
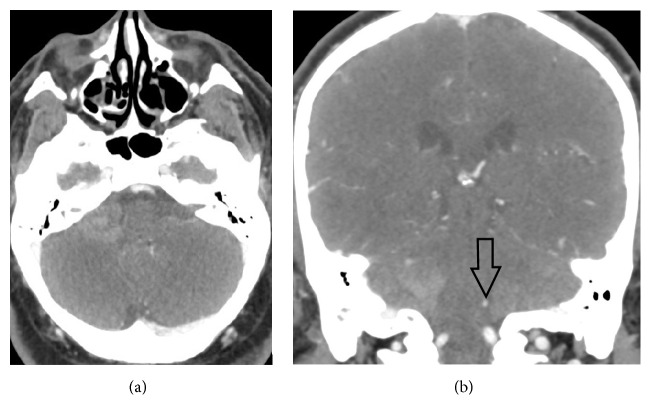
Axial (a) and coronal (b) CT angiography (CTA) of the head in the arterial phase demonstrates unchanged hemorrhage in the region inferior right cerebellar peduncle, likely intraparenchymal, with extension into the fourth ventricle without evidence of aneurysm or vascular malformation. The right posterior inferior cerebellar artery (PICA) is not well seen (arrow in (b) indicates the normal left PICA) and may be compressed by surrounding hemorrhage, involved with vasospasm or thrombosed.

A head computed tomography (CT) without contrast demonstrated hemorrhage in the region of the inferior right cerebellar peduncle, which appeared intraparenchymal, with extension into the fourth ventricle (Figure 1). A head CT angiography (CTA) with contrast was then performed, which did not reveal an aneurysm or vascular malformation (Figure 2). The right posterior inferior cerebellar artery (PICA) was not well seen and was thought to be compressed by surrounding hemorrhage, involved with vasospasm or thrombosed.

**Figure 3:**
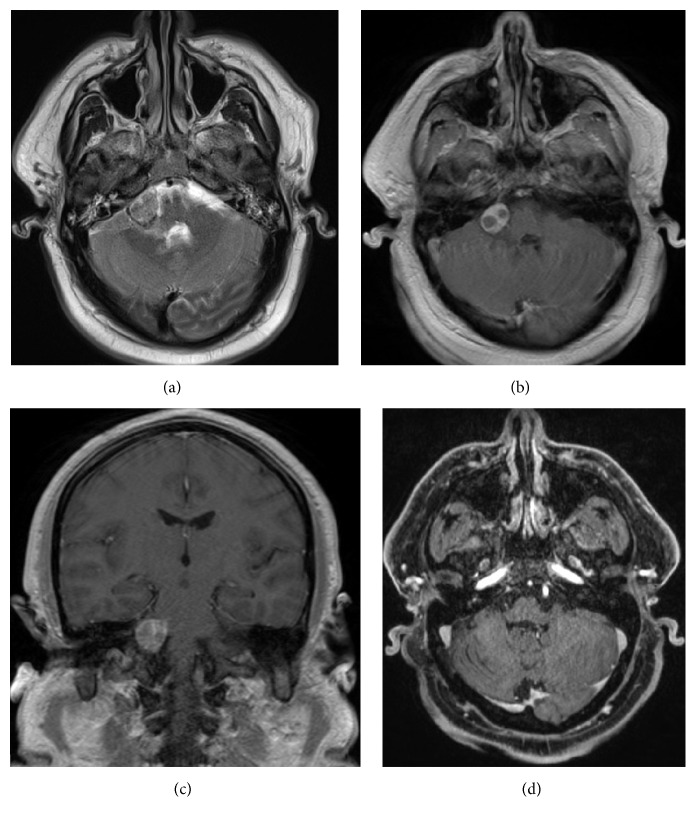
Axial T2-weighted (a) as well as axial (b) and coronal (c) T1-weighted contrast-enhanced magnetic resonance imaging (MRI) of the brain demonstrates an enhanced right cerebellopontine angle mass underlying the hemorrhage in the right cerebellopontine angle with central areas of nonenhancement. One year after resection, axial 3D T1-weighted contrast-enhanced MRI of the brain (d) demonstrates interval postsurgical changes of right retromastoid craniotomy for resection of the right cerebellopontine angle tumor without evidence of residual tumor.

Magnetic resonance imaging (MRI) of the brain demonstrated an enhanced right cerebellopontine angle mass underlying the hemorrhage in the right cerebellopontine angle (Figures 3(b) and 3(c)). A cleft of T2 hyperintensity between the mass and the medulla representing cerebrospinal fluid established that the mass and hemorrhage were actually extra-axial (Figure 3(a)). Catheter angiography was also performed, which demonstrated a tumor blush without aneurysm or vascular malformation (images not shown). In addition, as metastasis was included in the differential diagnosis, a whole-body PET/CT was also attempted to make an evaluation for a potential primary malignancy and possible additional lesions elsewhere; however, the study was not successful due to the patient's claustrophobia.

**Figure 4:**
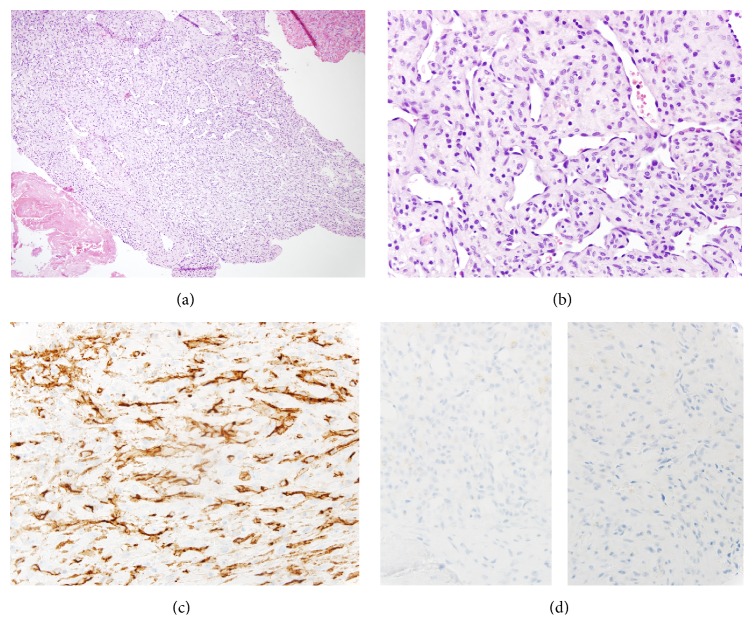
Hemangioma of the cerebellopontine angle. (a)-(b) Microscopically, the tumor cells are cytologically bland with vascular proliferation (H&E, (a) ×100; (b) ×400). (c)-(d) Immunohistochemistry, the tumor cells are positive for CD31 ((c), ×400) as well as negative for inhibin ((d) left, ×400) and EMA ((d) right, ×400). The cells are also positive for CD34 and negative for GLUT-1 (not pictured).

The patient subsequently underwent resection of the mass. Histologic evaluation of the mass showed cytologically bland tumor cells with vascular proliferation, which were positive for cluster of differentiation (CD) 31 and CD34 as well as negative for inhibin, glucose transporter 1 (GLUT-1), and epithelial membrane antigen (EMA), consistent with a capillary hemangioma (Figure 4). Finally, brain MRI performed one year later demonstrated interval resection of the right cerebellopontine angle tumor without evidence of residual tumor (Figure 3(d)).

## 2. Discussion

A capillary hemangioma is a benign tumor consisting of an abnormal overgrowth of tiny blood vessels. They appear within the first 6 months of age, grow rapidly until about 12 months of age, and then usually undergo complete spontaneous regression by 5 years of age [1]. Capillary hemangiomas of the skin are one of the most common tumors of infancy, with an estimated frequency of 10% within the first year of life [2]. Conversely, capillary hemangiomas rarely occur within the intracranial vault. Mirza et al. cataloged 24 case reports of ICH appearing in the literature [3]. Furthermore, only 10 cases of ICH have been reported in adults, and only three cases have been reported in adult males [3]. Two of the three cases in adult males involved the orbits, and all three were supratentorial [3]. To the best of our knowledge, this is the first ever reported case of an infratentorial capillary hemangioma in an adult male.

While it is extremely unlikely for an ICH to be prospectively diagnosed based on imaging alone given its rarity and lack of pathognomonic features, it can be suggested based on its typical imaging appearance. The enhancement pattern of an ICH is as expected: an avidly enhanced mass, given its vascular nature. Likewise, it also has the capability to bleed spontaneously as manifested in our case by hyperdensity in the region of the inferior right cerebellar peduncle on the initial head CT. While the hemorrhage appeared intra-axial on CT, it was confirmed to be extra-axial on MRI. This is in keeping with the fact that most intracranial capillary hemangiomas are extra-axial [3]. Intracranial capillary hemangiomas are usually enhanced homogenously, though that was not seen in our case, which may relate to intratumoral necrosis or hemorrhage [3]. Lastly, while not seen in our case, there can be areas of T2 hypointensity signifying flow voids.

Pathologic diagnosis was aided by CD31 and CD34, which highlight vascular architecture. Both immunochemical stains were positive in this case as is required for diagnosis of a hemangioma. Additionally, while GLUT-1 is typically positive in infantile and juvenile hemangiomas, it was negative in this case given the patient's adult age.

There are a number of other diagnoses to consider for an enhanced mass in the cerebellopontine angle in an adult. Although the most common mass in this location would be a schwannoma, these rarely hemorrhage or have intratumoral hemorrhage and are typically hypovascular to avascular on catheter angiography [4]. The main diagnostic considerations are hemangioblastoma (particularly in the absence of homogenous enhancement), metastatic disease, ependymoma, or a choroid plexus tumor. An exophytic glial neoplasm is unlikely in the setting of an apparently hemorrhagic extra-axial mass. A meningioma is unlikely given the intratumoral hemorrhage and heterogeneous enhancement, as well as the absence of a dural tail. While our patient did not have a known primary malignancy, the possibility of metastatic disease appears to be the most common reason to pursue further workup and surgical resection in patients who were ultimately diagnosed with ICH [5].

In conclusion, ICH is a rare entity. We presented the first ever reported case of a capillary hemangioma in the posterior fossa of an adult male. As such, a capillary hemangioma could be considered in the differential diagnosis for an enhanced extra-axial mass in the posterior fossa, particularly in the setting of spontaneous hemorrhage.
